# PD64 Modeling Clinical And Economic Impact Of Integral, Transversal, And Multidisciplinary Management Of Aortic Stenosis In A Catalan Hospital

**DOI:** 10.1017/S0266462324003210

**Published:** 2025-01-07

**Authors:** Carla Fernandez-Barcelo, Bàrbara Vidal Hagemeijer, Ismail Abbas, Marc Trilla, Marta Sitges Carreño, Laura Sampietro-Colom

## Abstract

**Introduction:**

A program for integral, transversal, and multidisciplinary management of aortic stenosis (MITMEVA) is being implemented at the Clinic Barcelona University Hospital (CBUH) to provide adequate treatment for patients with aortic stenosis (AS). Eleven actions at different care points were implemented (e.g., awareness raising for the general population, a single entry path for patient referral, prehabilitation and rehabilitation, and a risk-sharing agreement). Preliminary results are presented.

**Methods:**

A before-and-after implementation study was conducted with 131 patients under MITMEVA and 131 matched (for treatment, New York Heart Association classification, sex, age, and referral place) historical controls. Data were collected on resources used and quality of life and to calculate several key performance indicators (KPIs) (e.g., knowledge improvement in citizens, time from diagnosis to treatment, and patient involvement and satisfaction) for each implemented action. A descriptive analysis of KPIs and a Markov model were performed to simulate clinical and economic outcomes for patient health states over time after the first year until the tenth year after intervention.

**Results:**

The MITMEVA program increased quality-adjusted life-years by 1.78 (p=0.011) and reduced time from referral to first hospital visit by 24.7 percent (p=0.05), hospital complications by 19.7 percent (p=0.05), mean conventional ward stay from 12.8 to 8 days (p=0.01), and mean intensive care unit stay from 9.75 to 4.25 days, although the latter difference was not statistically significant (p=0.139). The mean cost per patient was reduced from EUR7,573.27 per patient to EUR6,024.61 per patient (p=0.01). The MITMEVA program was a dominant strategy. There was a 46 percent increase in correct AS symptom identification after delivering training on AS to the general population.

**Conclusions:**

Integrated care approaches can potentially improve patient continuum of care if the strategies are deployed in a multidisciplinary and transversal way across healthcare actors. The MITMEVA program significantly improved clinical and economic outcomes and organization of care, benefiting patients and clinicians. Applying health technology assessment methods to such innovative projects can help prove the value of organizational innovations.

